# Using automated software evaluation to improve the performance of breast radiographers in tomosynthesis screening

**DOI:** 10.1007/s00330-023-10457-x

**Published:** 2023-11-29

**Authors:** Gisella Gennaro, Letizia Povolo, Sara Del Genio, Lina Ciampani, Chiara Fasoli, Paolo Carlevaris, Maria Petrioli, Tiziana Masiero, Federico Maggetto, Francesca Caumo

**Affiliations:** Unit of Breast Radiology, Department of Imaging and Radiotherapy, Veneto Institute of Oncology (IOV), IRCCS, via Gattamelata 64, 35128 Padua, Italy

**Keywords:** Mammography, Breast, Early detection of cancer, Quality improvement, Image processing (computer-assisted)

## Abstract

**Objective:**

To improve breast radiographers’ individual performance by using automated software to assess the correctness of breast positioning and compression in tomosynthesis screening.

**Materials and methods:**

In this retrospective longitudinal analysis of prospective cohorts, six breast radiographers with varying experience in the field were asked to use automated software to improve their performance in breast compression and positioning. The software tool automatically analyzes craniocaudal (CC) and mediolateral oblique (MLO) views for their positioning quality by scoring them according to PGMI classifications (perfect, good, moderate, inadequate) and checking whether the compression pressure is within the target range. The positioning and compression data from the studies acquired before the start of the project were used as individual baselines, while the data obtained after the training were used to test whether conscious use of the software could help the radiographers improve their performance. The percentage of views rated perfect or good and the percentage of views in target compression were used as overall metrics to assess changes in performance.

**Results:**

Following the use of the software, all radiographers significantly increased the percentage of images rated as perfect or good in both CCs and MLOs. Individual improvements ranged from 7 to 14% for CC and 10 to 16% for MLO views. Moreover, most radiographers exhibited improved compression performance in CCs, with improvements up to 16%.

**Conclusion:**

Active use of a software tool to automatically assess the correctness of breast compression and positioning in breast cancer screening can improve the performance of radiographers.

**Clinical relevance statement:**

This study suggests that the use of a software tool for automatically evaluating correctness of breast compression and positioning in breast cancer screening can improve the performance of radiographers on these metrics, which may ultimately lead to improved screening outcomes.

**Key Points:**

*• Proper breast positioning and compression are critical in breast cancer screening to ensure accurate diagnosis.*

*• Active use of the software increased the quality of craniocaudal and mediolateral oblique views acquired by all radiographers.*

*• Improved performance of radiographers is expected to improve screening outcomes.*

## Introduction

Breast cancer is a major public health concern, and early detection through screening is crucial for improved outcomes [1]. Mammography is the most widely used imaging modality for breast cancer detection, but its accuracy can be impacted by factors such as breast positioning and compression [2–5]. Proper breast positioning in mammography is crucial for several reasons. It helps to ensure that all areas of the breast are properly captured in the image [3], leading to better visualization of any potential abnormalities, and reduces the amount of radiation [6] required to produce a high-quality image. In addition, proper positioning can make the mammography experience better for the patient, reducing discomfort. More importantly, correct breast positioning helps ensure that the radiologist is able to obtain accurate, detailed images that are essential for accurate diagnosis [7].

Digital breast tomosynthesis (DBT) is a newer form of mammography that uses multiple projection images to create a three-dimensional reconstruction of the breast, and has been shown to improve cancer detection rates compared to conventional mammography [8–12]. However, as requirements of breast positioning and compression are unchanged with DBT, the accuracy of DBT is also dependent on proper breast positioning and compression.

The quality of breast positioning during mammography or DBT can be assessed using visual methods such as the “PGMI Image Evaluation System” published in 1994 by the National Health System Breast Screening Program of the UK [13] or the criteria included in the Mammography Quality Control Manual published by the American College of Radiology in 1999 [14], or derived visual methods [15]. Visual assessment methods have two main limitations: one is the need for sampling, as visual assessment is time-consuming, and the other is inter-observer variability [16].

In recent years, automatic software tools have been developed to evaluate the correctness of breast positioning and compression during mammography or tomosynthesis [5, 17–21]. Systematic collection of positioning and compression data can be used as a quality assurance tool, which is much more powerful than other visual methods that require manual completion of a form and inevitably rely on small image samples [15, 22, 23].

The purpose of this study was to investigate whether the informed use of automated software to assess the quality of positioning and compression can improve the individual performance of breast radiographers by increasing the number of high-quality mammograms.

## Materials and methods

### Study population

This retrospective longitudinal analysis of prospective cohorts aimed to evaluate the performance of six breast radiographers in breast positioning and compression during tomosynthesis screening. The study data were obtained from the RIBBS (Risk-Based Breast Screening) study, a prospective screening trial aiming to assess the effectiveness and sustainability of a personalized screening model for young women based on individual breast density and breast cancer risk.

The RIBBS study invited women aged 45 residing in an area close to our Institution, willing and able to provide written informed consent and comply with scheduled visits, examinations, and other procedures. All accepting women underwent DBT, which was independently evaluated by two breast radiologists (double reading). Quantitative breast density was measured from the DBT images, and individual risk was assessed using the Tyrer-Cuzick risk model [24]. Imaging protocols and intervals of the subsequent screening rounds were defined based on the breast density and risk category (low, intermediate, high). Exclusion criteria comprised a personal history of breast cancer, known BRCA or PALB2 mutations, pregnancy or breastfeeding, and disorders incompatible with protocol requirements and follow-up. Further details about the RIBBS study can be found at ClinicalTrials.gov (NCT05675085). The study is currently ongoing for subsequent screening rounds, and enrolled women continue to be rescreened with personalized protocols determined by their breast density and individual risk until they reach the age of 50. The study received ethics committee approval, and all 10,269 recruited 45-year-old women signed an informed consent form, permitting the use of their data for any subsequent retrospective analysis. As a result, the current study was considered exempt from review by the Ethics Committee.

In this quality improvement project, a retrospective evaluation was conducted of breast positioning and compression parameters measured with an automated software tool from two DBT data sets belonging to the RIBBS study. All radiographers in service at the beginning of the project, with breast imaging experience ranging from 0 to more than 25 years, agreed to participate. Acceptance of this diversity of work experience was a key aspect of our project, which aimed to provide a flexible and personalized learning experience for each radiographer.

Two Hologic DBT units were used to acquire all screening examinations. Each machine captured 15 projection images over a 15° arc (± 7.5°) and reconstructed tomosynthesis planes that were sampled every 1 mm in the *z* direction. Bilateral DBT in two views (craniocaudal—CC, and mediolateral oblique—MLO) with synthetic mammography (SM) reconstructed from DBT was used for the screening protocol [8, 9, 11, 25].

### Automated assessment of breast positioning and compression

In this study, breast positioning and compression parameters were evaluated using Volpara Analytics (Volpara Health). The software systematically analyzes CC and MLO images acquired by different modalities (digital mammography, tomosynthesis, and contrast-enhanced mammography) and collects different types of data, including volumetric breast density, breast compression and positioning parameters, exposure data, and radiation dose in a single cloud-based database. The software employs the Volpara TruPGMI clinical function, which assesses breast positioning based on international best practices, such as the UK PGMI standard, and the adapted PGMI criteria from Australia, New Zealand, Norway, and the American College of Radiology [26]. This method establishes four positioning metrics for CC views and seven for MLOs, categorizing outcomes into four classes (perfect—P; good—G; moderate—M; inadequate—I), as reported in Table 1. Furthermore, the software calculates the percentage of images classified as perfect or good (% P + G), serving as a comprehensive measure of positioning accuracy.

**Table 1:** Metrics evaluated for CC and MLO views by the automated software and related PGMI scores

View	Metric	Description	P	G	M	I
CC	Nipple in profile	The nipple protrudes from the skin line	X	O	O	O
	Nipple midline	The nipple is central and perpendicular in the breast, and is not exaggerated either medially or laterally by more than 5 degrees	X	O	O	O
	CC PNL	The CC PNL is no less than 1 cm shorter than the MLO PNL	X	X	O	O
	No cutoff	The breast tissue is not cut off, either medially or laterally	X	X	X	O
MLO	Nipple in profile	The nipple protrudes from the skin line	X	O	O	O
	IMF visible	The IMF is in view and open	X	O	O	O
	Pec to PNL Met	The pectoral muscle extends inferiorly to at least 1 cm above the level of the PNL	X	X	O	O
	Adequate Pec	The vertical length is at least 1/3 of the breast height and the pectoral muscle angle is between 10 degrees (narrow pectoral muscle) and 20 degrees (wide pectoral muscle)	X	X	O	O
	Pectoral shape	The pectoral muscle is either convex or straight	X	O	O	O
	No Pec skinfolds	No linear skin folds are detected on the pectoral muscle	X	O	O	O
	No cutoff	The breast tissue is not cut off inferiorly	X	X	X	O
X = must be met O = may or may not be met						

*CC*, craniocaudal; *MLO*, mediolateral oblique; *PNL*, pectoral muscle to nipple line; *IMF*, inframammary fold; *Pec*, pectoral muscle

In addition, the software determines the compression pressure applied to the breast by dividing the compression force reported in the DICOM header by the software-derived contact area of the breast with the compression plate. The target compression pressure range was set between 7 and 15 kPa [19, 27]; pressure values below or above that range are classified as low and high compressions, respectively. The percentage of images with compression in the target range is provided as a metric of compression quality.

The software presents breast positioning and compression performance metrics in interactive dashboards accessible through a web browser. Radiographers can review and monitor their performance, identifying areas for improvement by comparing their metrics against aggregate measures from their peers within the institution and other Volpara Analytics users globally (over 5200 radiographers with more than 85 million images from several countries) [27]. The software interface ensures that radiographers can exclusively access data generated from the images they acquired themselves, without access to data from other radiographers.

### Quality improvement method

The software was available to the breast radiographers since 2019 via the exam room computer, but they were not actively encouraged to use it until 2022. In 2022, a quality improvement project was initiated to support the individual improvement of radiographers, using the software. The project was led by a medical physicist, the lead radiographer, and a thesis student (undergraduate radiographer).

A large dataset of DBT screening studies acquired by five of the six radiographers participating in the study served as the “baseline” for compression and positioning data (“BEFORE training” dataset, Sep–Dec 2021). Individual metrics were compared with the median of the global distribution of software users [27]. Results from this baseline were shared with the five radiographers who produced them and a newly graduated colleague who joined the team at the start of the project.

A training course was organized to familiarize all radiographers with software functionalities, and comprehensive assessment and open discussions of positioning and compression criteria were conducted, using real sample cases. Radiographers were encouraged to actively engage with the software interface individually to evaluate their data. This included a combination of individual use of the software, which could vary among radiographers, and regular participation in monthly face-to-face meetings with a thesis student. During these meetings, radiographers received comprehensive assistance in analyzing their data, identifying areas for improvement, and tracking progress.

To provide context, a 2-month training period was scheduled in January and February 2022 to allow radiographers to familiarize themselves with the software before the test period began in March 2022. The testing period was designed to facilitate improvements in individual metrics that were below the global median.

Regular face-to-face meetings with the thesis student played a key role in ensuring that all radiographers remained well informed about changes in their performance and were equipped to effectively address their areas for improvement. Throughout the study, the purpose of using the software was to promote a collaborative environment among radiographers, emphasizing teamwork over competition.

In essence, the “active use of the software” in this study represented a holistic approach, combining individual interactions with the software and personalized support provided during monthly meetings by the thesis student to facilitate performance analysis, improvement, and continuous evaluation of progress.

**Fig. 1:**
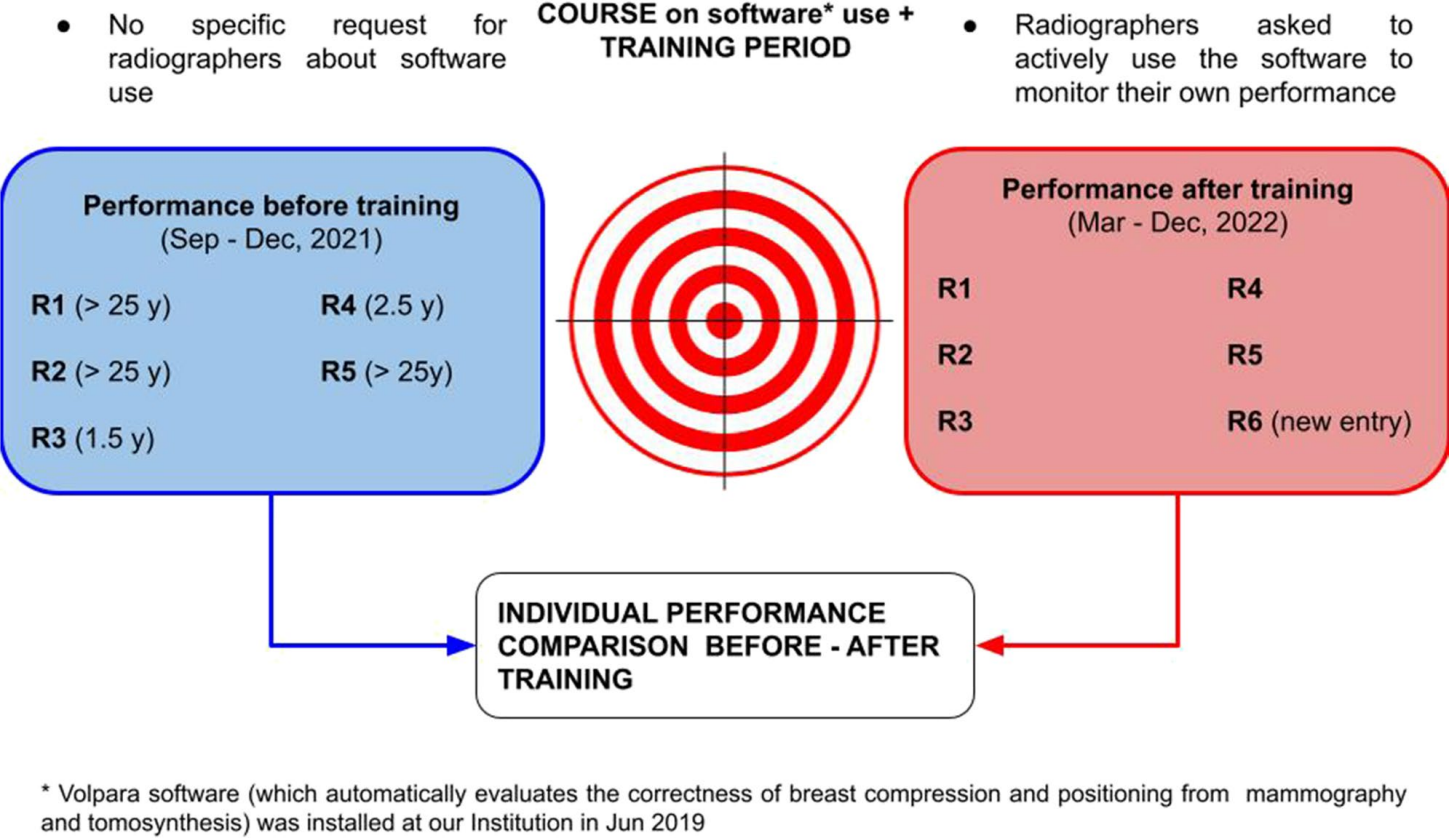
Schematic representation of the study design. The evaluation of the radiographer’s performance in breast compression and positioning was conducted using a set of screening data acquired before software training. Baseline performance was established and used as a reference point. Then, radiographers participated in a course to establish individual improvement goals and a training period to familiarize themselves with the automated software. Finally, post-training screening data were compared with baseline data to assess the improvement of the software aid

The dataset used to test the impact of using the software consisted of screening images acquired between March and December 2022 from the five radiographers who contributed to the baseline and the newly joined colleague (“AFTER training” dataset). Figure 1 illustrates the experiment’s schematics.

### Statistical analysis

Individual changes in positioning metrics and compression performance were analyzed, and the differences in the proportions of images rated perfect or good before and after the training period for each radiographer were tested using a two-sample *Z* test of proportions. A *p*-value of less than 0.05 was considered statistically significant. Statistical analysis was performed using OriginPro 2020b (OriginLab Corporation) and MedCalc v. 20.216 (MedCalc Software Ltd).

The study was conducted in adherence to SQUIRE (Standards for QUality Improvement Reporting Excellence) guidelines v 2.0 [28].

## Results

### Study population

The study population included 2407 women in the pre-software training cohort and 3986 in the post-software cohort. The characteristics of the study population are given in Table 2 in terms of median values and their 95% confidence intervals (95% CI), and include the women’s age, breast thickness and compression force extracted from the DICOM image header, and breast volume, volumetric breast density (VBD), contact area, and compression pressure calculated by the Volpara software.

**Table 2:** Study population description: overall age, compressed breast thickness, breast volume, compression force, contact area, compression pressure, and volumetric breast density (VBD) have been provided (median values and interquartile range (IQR))

Parameter	Median	IQR
Women age (y)	46	46–47
Breast thickness (mm)	52	42–63
Breast volume (cm^3^)	609.9	381.7–963.2
Force (N)	89.0	76.2–103.5
Contact area (dm^2^)	0.868	0.638–1.140
Pressure (kPa)	10.13	7.96–13.33
VBD (%)	12.0	6.7–19.2

Among the six radiographers engaged in the project, three possessed more than 25 years of experience in mammography and tomosynthesis. Of the other three, two had experience of 2.5 years and 1.5 years, respectively. The last member started her role in breast radiology from January 2022. In total, 9609 views (4815 CCs and 4794 MLOs) were analyzed for the before training period, and 15,853 views (7942 CCs and 7911 MLOs) for the after training period. Table 3 provides details on the breast imaging experience of each radiographer at the beginning of the study and the total number of DBT screening studies and views. Each screening study included two CCs and two MLOs; however, some discrepancies between number of CCs and MLOs are caused by some software exclusions because of possible implant presence in the field of view, mosaic in case of extremely large breasts, medial–lateral or lateral-medial views.

**Table 3:** Radiographers’ years of experience in mammography and tomosynthesis at the start of the study and the total number of individually acquired DBT studies and views (a study consists of two CCs and two MLOs)

Radiographer ID	Experience in mammography/tomosynthesis (y)	BEFORE software training dataset (n° of studies and CC/MLO views)	AFTER software training dataset (n° of studies and CC/MLO views)
R1	> 25	Studies: 588	Studies: 762
		CCs: 1178	CCs: 1524
		MLOs: 1174	MLOs: 1521
R2	> 25	Studies: 558	Studies: 767
		CCs: 1118	CCs: 1537
		MLOs: 1117	MLOs: 1531
R3	1.5	Studies: 483	Studies: 846
		CCs: 964	CCs: 1691
		MLOs: 953	MLOs: 1686
R4	2.5	Studies: 369	Studies: 484
		CCs: 736	CCs: 966
		MLOs: 734	MLOs: 957
R5	> 25	Studies: 409	Studies: 500
		CCs: 819	CCs: 999
		MLOs: 816	MLOs: 999
R6	0	n.a	Studies: 612
			CCs: 1225
			MLOs: 1217
Total	avb	Studies: 2407	Studies: 3986
		CCs: 4815	CCs: 7942
		MLOs: 4794	MLOs: 7911

*CC*, craniocaudal; *MLO*, mediolateral oblique

### Baseline performance

**Fig. 2:**
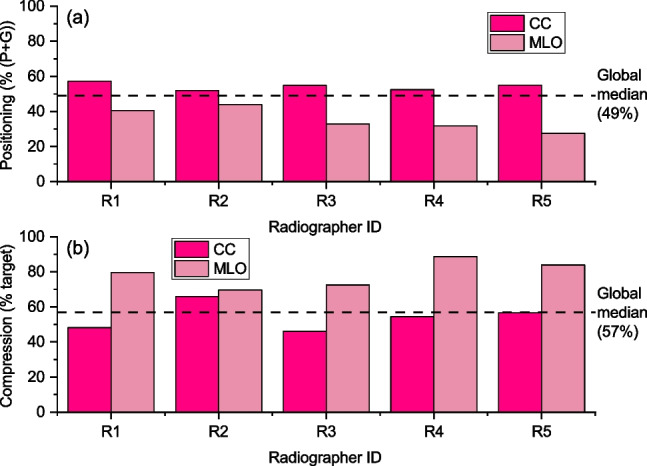
**a** Baseline positioning performance for five of the six radiographers involved in the study; positioning performance is represented by the percentage of images scored perfect or good according to the PGMI method. **b** Baseline compression performance for five of the six radiographers involved in the study; compression performance is represented by the percentage of images that fall within target compression. Results are shown separately for CC and MLO views. Global median values for both positioning and compression metrics, used as benchmarks, are also provided

In Fig. 2, the before training performance of five radiographers who contributed to the study is presented in terms of (a) percentage of CCs and MLOs scored perfect or good, and (b) percentage of CCs and MLOs within the target compression. In addition, the median performance of all software users is shown as a benchmark. It is worth noting that already from baseline all five radiographers performed better in CC positioning than in MLOs, exceeding the median only for CC views. In contrast, all demonstrated better compression performance for MLOs than for CCs, with results well above the median value. Only R2 achieved a percentage of CCs in target compression above the overall median.

In order to determine individual practical improvement goals, all baseline positioning and compression metrics were evaluated for the CC and MLO views, shown in Table 4, along with the global medians as reference values. The percentage of images (CCs and MLOs) that met each positioning and compression metric was reported for each radiographer. Values below the global median were highlighted in bold.

**Table 4:** Percentage of baseline images (CCs and MLOs) that met each positioning and compression metric for each radiographer at the baseline (before training dataset). Values below the global median are highlighted in bold

View	Metric	R1 (%)	R2 (%)	R3 (%)	R4 (%)	R5 (%)	Global median (%)
CC	Nipple in profile	96.6	97.2	94.5	94.8	96.1	79.0
	PNL met	68.2	72.9	70.1	80.8	69.8	68.0
	No cutoff	98.9	100.0	99.6	99.2	99.6	98.0
	Nipple midline	52.8	**36.8**	47.5	**30.8**	46.2	46.0
	Target compression	**48.2**	66.0	**46.1**	**54.5**	**56.6**	58.0
MLO	Nipple in profile	92.8	96.9	85.0	94.4	95.2	82.0
	IMF visible	39.9	40.6	**22.7**	**26.5**	**23.8**	36.0
	Pec to PNL Met	73.6	82.1	67.7	74.0	65.4	65.0
	Adequate Pec	93.6	95.3	92.6	94.0	94.7	93.0
	No cutoff	**98.9**	100.0	99.4	99.6	99.5	99.0
	Pec shape	**56.3**	**63.7**	**51.0**	**68.8**	**60.5**	76.0
	No Pec skinfolds	96.8	97.7	99.5	99.3	98.2	96.0
	Target compression	79.6	69.6	72.5	88.6	83.9	55.0

*CC*, craniocaudal; *MLO*, mediolateral oblique; *PNL*, pectoral muscle to nipple line; *IMF*, inframammary fold; Pec, pectoral muscle

### Performance improvement

After the training period, all six radiographers (the five for which baseline performance was reported, plus the new entry) started to actively use the software to improve their positioning and compression performance in screening.

**Fig. 3:**
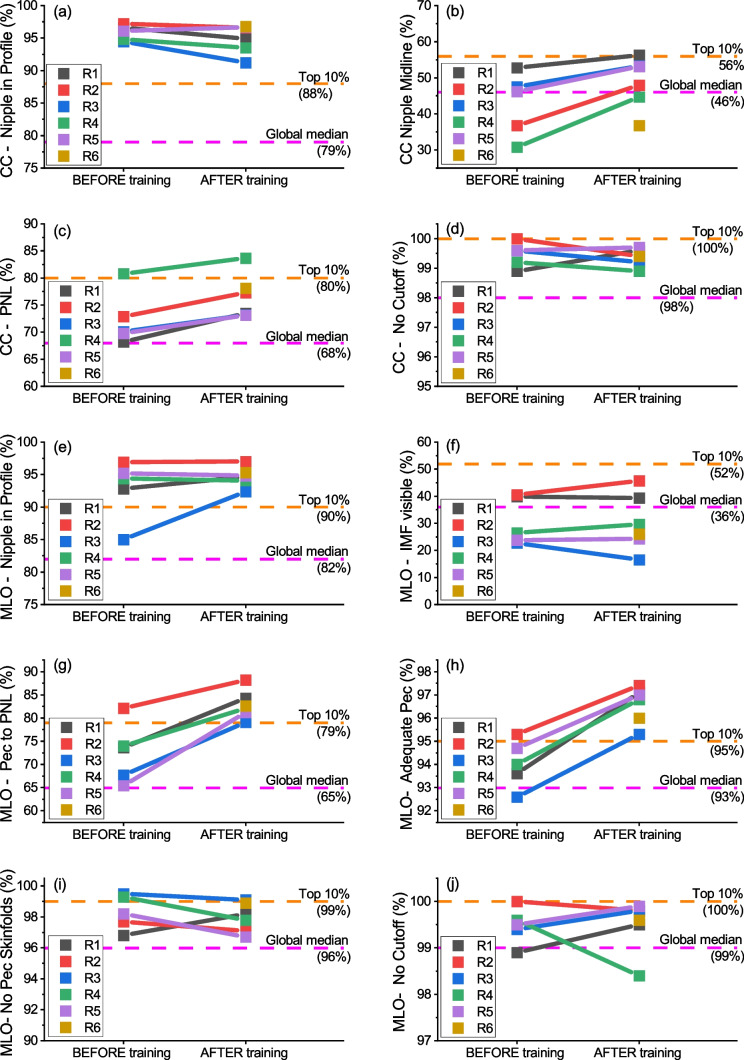
Comparison between baseline and final datasets after an active software use for each breast positioning metric and each breast radiographer. **a** “Nipple in profile” metric for CC views; **b** “Nipple midline” metric for CC views; **c** “PNL met” metric for CC views; **d** “No cutoff” metric for CC views; **e** “Nipple in profile” metric for MLO views; **f** “IMF visible” metric for MLO views; **g** “Pec to PNL” metric for MLO views; **h** “Adequate Pec” metric for MLO views; **i** “No Pec skinfolds” metric for MLO views; **j** “No cutoff” metric for MLO views

Figure 3 displays individual changes in specific positioning metrics, along with the median and top 10% values from the global distribution of software users. The criterion “Pec shape” for MLO views was excluded from this representation because it was not part of the original PGMI method. Although it is a useful metric, it is not necessary to achieve a score of “Good” and is strongly correlated with two other critical metrics, “Pec to PNL Met” and “Adequate Pec.” In addition, this criterion was excluded for graphical purposes.

Considering the “No cutoff” metric as the most crucial factor (necessary to avoid rating the image inadequate), it is observed that only one radiographer (R1) improved this criterion in CC views, while three radiographers (R1, R3, R5) did so in MLO views. Nonetheless, the “No cutoff” metric for CC views fell between the median and top 10% of the global distribution for all radiographers, including the new one, as did the metric for MLO views, except for R4, who lowered the percentage of no-cutoff images below the global median compared to the baseline value. Three other important metrics were “PNL Met” for CC views and “Pec to PNL” and “Adequate Pec” for MLO views. Each radiographer improved all these metrics, surpassing the top 10% line for MLOs. The “Nipple in profile” metric showed improvement for MLO views by most radiographers, while for CC views, it already exceeded the top 10% mark. The “Nipple midline” metric improved for CC views by all radiographers, and MLO inframammary fold visibility was enhanced by the majority of them. Lastly, the “No Pec skinfold” metric remained relatively stable for all radiographers, falling between the global median and top 10% of software users.

It is noteworthy that the young radiographer who joined the breast radiology team after graduation and actively used the software from the beginning performed equivalent to more experienced colleagues and even exceeded the overall medians in some metrics.

**Fig. 4:**
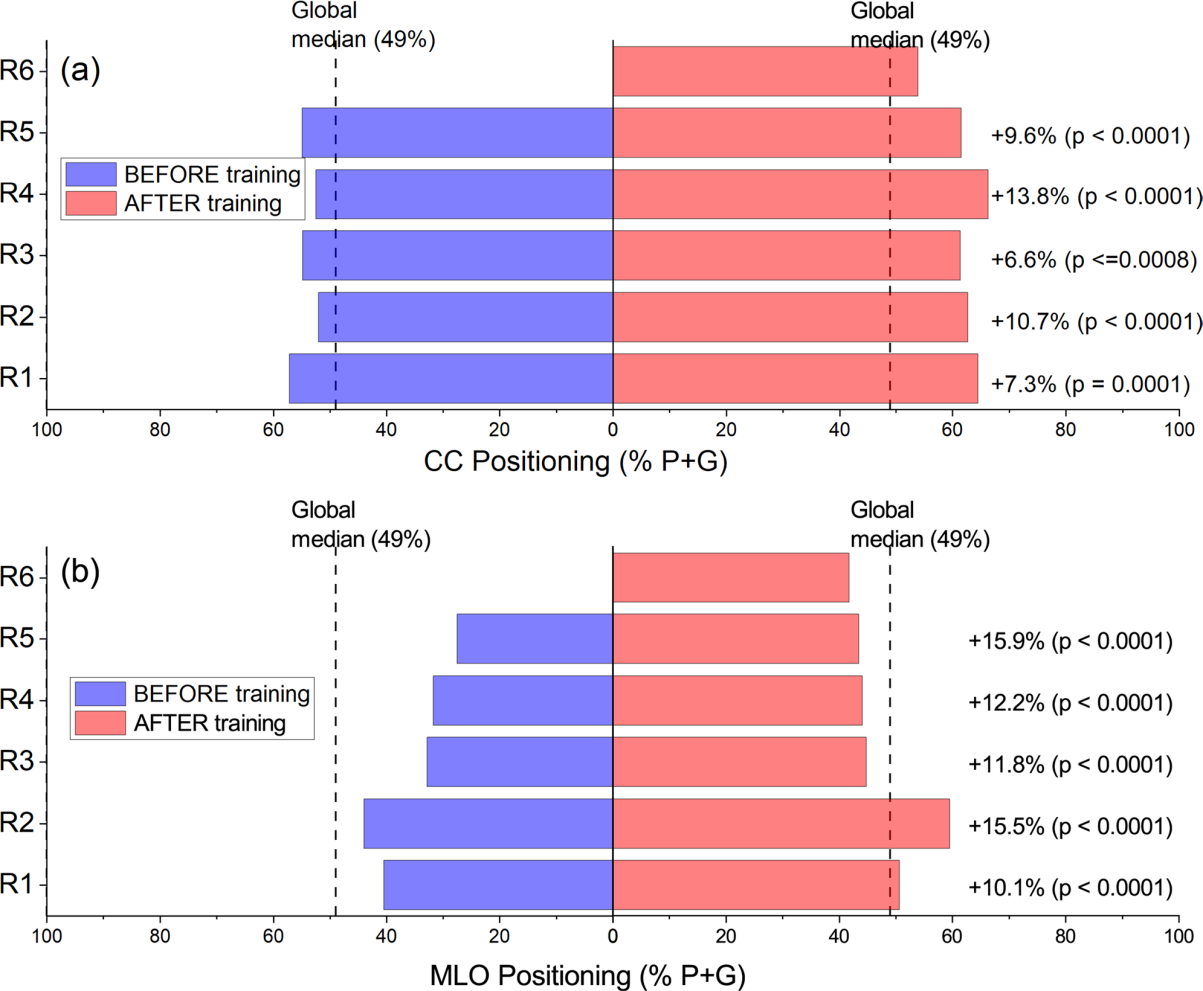
**a** Comparison between percentage of CC images rated perfect or good in the baseline (in blue) and in the final dataset (in red) after starting the active software use. **b** Comparison between percentage of MLO images rated perfect or good in the baseline and in the final dataset after starting the active software use

Figure 4 shows that all changes in individual positioning metrics affected the proportion of images rated as perfect or good. The active use of the software tool significantly increased the proportion of images rated as perfect or good (in both CCs and MLOs) for each radiographer. Individual improvements ranged from 7 to 14% for CC and 10 to 16% for MLO views.

**Fig. 5:**
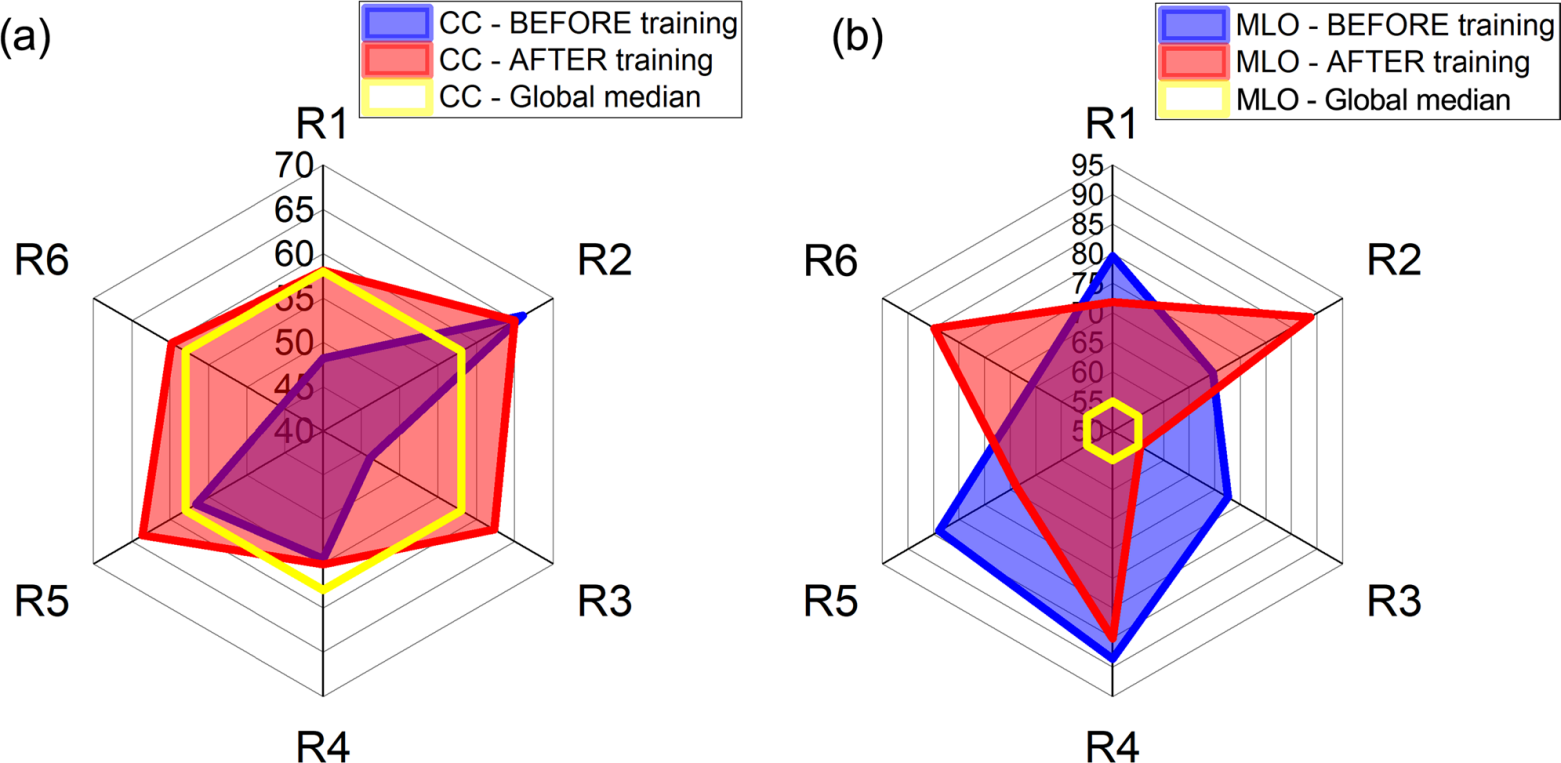
Radar plots showing the percentage of images within the target compression in the baseline dataset (in blue) and final dataset (in red) after the active software use for (**a**) CC views and (**b**) MLO views. The global median values are shown in yellow to provide a benchmark

Radar plots in Fig. 5 illustrate the percentage of images within the target compression in periods before and after training for active use of the software.

Overall, there was a rise in the number of correctly compressed CC views (up to 16%), with the majority of radiographers performing equally or better than the global median. When it came to MLO compression, only R2 showed improvement compared to the baseline, but all radiographers except R3 demonstrated significantly better MLO compression than the global median of software users.

## Discussion

The present study aimed to evaluate the impact of using automated software that assesses the correctness of breast compression and positioning based on the PGMI method on the performance of breast radiographers. Results indicated that after software training and active use, all five evaluated breast radiographers showed an increase in the percentage of images rated as perfect or good, as well as in the percentage of images achieving the target compression. The sixth radiographer, who began using the software from the start, performed above the median for CC views and nearly as well as more experienced colleagues for MLO views. These findings indicate that an active and informed use of automated software which can assess the correctness of breast compression and positioning based on the PGMI method can improve radiographer performance regardless of experience level in mammography or tomosynthesis screening.

Regular updates and interactions with the thesis student played a crucial role in helping radiographers identify areas for improvement and make informed adjustments to their positioning and compression techniques.

The project was designed to support individual improvements, focusing on each radiographer’s specific needs and areas for development, rather than aiming for a standardized level of performance. Taking a data-driven approach, radiographers were encouraged to concentrate on specific aspects of their practice most relevant to their professional growth. The flexibility of the project and the personalized learning experience proved useful in helping radiographers to effectively identify and address their weaknesses.

Other factors, such as breast size, breast density, and radiographers’ fatigue, were intentionally excluded from the study, as the primary objective was to verify the effectiveness of the software tool in improving individual radiographer performance, rather than engaging in a speculative analysis of factors associated with breast mispositioning or under/over-compression.

The study by Pal et al demonstrated that visual evaluation of positioning criteria can lead to an improvement in mammographic positioning skills among radiographers [23]. However, visual assessment has limitations, particularly in the time-consuming process of evaluating a limited number of mammography/tomosynthesis examinations, potentially reducing the strength of performance evaluation before and after training. Moreover, visual assessment often lacks consensus among radiographers and radiologists in interpreting positioning criteria, as highlighted by Spuur [29] and Taylor [30].

In a study by Waade et al, 156 screening mammography exams were visually evaluated using the PGMI method by two experienced radiographers, and the resulting positioning criteria were compared to those generated by the software used in our study [17]. The researchers observed substantial to almost perfect agreement between the software and radiographers for certain criteria, such as nipple in profile and PNL met in CC views, and slight to moderate agreement for others, such as adequate Pec and IMF visible in MLO views. Overall, they found that the agreement between radiographers was better than that between radiographers and the software [17]. Picard et al conducted a similar study in which they compared the paired visual assessment of positioning quality by a radiographer and a radiologist with that provided by automated software. They found that while some disparities existed between the readers’ subjective assessments and the objective assessments, considerable agreement existed between readers and software regarding the overall assessment of positioning [31].

It is important not to interpret the disagreement between visual evaluation and software as a failure of the software tool itself. In order to achieve “the full visualization of breast from the pectoral muscle to the nipple” described in the PGMI, software needs to perform precise geometrical measurements, such as measuring the Pec to PNL distance in each of the CC and MLO views for their comparison. In contrast, visual evaluation by human readers works differently and does not rely solely on geometric measurements. Visual evaluation can be quite subjective and influenced by image aesthetics or personal preferences. Poor agreement levels have been demonstrated between expert readers on evaluation of the depiction of various features of the normal tissue [32]. Therefore, differences in the evaluations between the software and visual assessments do not necessarily indicate a problem with the software tool, but rather may indicate a difference in the techniques utilized by humans and software to verify the same positioning criteria.

The use of an automated software to systematically score mammography images according to the PGMI criteria provides a more consistent, efficient, and effective approach to evaluating the quality of breast positioning in mammography and tomosynthesis screening.

Limitations of the study include the small number of radiographers involved, all from a single center, which may limit the generalizability of the results to other settings or populations. In addition, the study evaluated the short-term impact of software use on positioning and compression performance, leaving open questions about the sustainability of the observed improvements over an extended period. Another limitation concerns the specific screening population of the study, which included only women aged 46–47, a selection dictated by screening population available at our institution. However, we emphasize that the study design and methodology could be applicable to any screening population.

In conclusion, this study suggests that the use of a software tool for automatically evaluating correctness of breast compression and positioning in breast cancer screening can potentially improve radiographer performance, which may ultimately lead to improved patient outcomes. It also reiterates that the path to improvement does not depend on technology alone, but is a collective endeavor rooted in human awareness and proactive engagement. The active participation of radiographers in analyzing their performance emerged as a catalyst for the observed improvements, underscoring the synergy between technology and human action in achieving excellence in breast radiography.

## Abbreviations

CC: Craniocaudal
DBT: Digital breast tomosynthesis
IMF: Inframammary fold
MLO: Mediolateral oblique
Pec: Pectoral muscle
PGMI: Perfect good moderate inadequate
PNL: Pectoral muscle to nipple line

## Declarations

### Guarantor

The scientific guarantor of this publication is Gisella Gennaro.

### Statistics and biometry

One of the authors has significant statistical expertise.

### Study subjects or cohorts overlap

There is no cohort overlap.

### Methodology

• retrospective longitudinal analysis of prospective cohorts

• observational

• performed at one institution
